# Three-dimensional evaluations of preoperative planning reproducibility for the osteosynthesis of distal radius fractures

**DOI:** 10.1186/s13018-021-02278-9

**Published:** 2021-02-12

**Authors:** Yuichi Yoshii, Takeshi Ogawa, Atsuo Shigi, Kunihiro Oka, Tsuyoshi Murase, Tomoo Ishii

**Affiliations:** 1 Department of Orthopaedic Surgery, Tokyo Medical University Ibaraki Medical Center, 3-20-1 Chuo, Ami, Inashiki, Ibaraki, 300-0395 Japan; 2 Department of Orthopaedic Surgery, University of Tsukuba Hospital, Tsukuba, Ibaraki, 305-8576 Japan; 3 Department of Orthopaedic Surgery, Osaka University Graduate School of Medicine, Suita, Osaka, 565-0871 Japan

**Keywords:** Computer-assisted orthopedic surgery, Computed tomography, Distal radius fracture, Osteosynthesis, Preoperative plan, Three dimensions

## Abstract

**Background:**

Three-dimensional preoperative planning was applied for the osteosynthesis of distal radius fractures. The objective of this study was to evaluate the reproducibility of three-dimensional preoperative planning for the osteosynthesis of distal radius fractures with three-dimensional reference points.

**Methods:**

Sixty-three wrists of 63 distal radius fracture patients who underwent osteosynthesis with three-dimensional preoperative planning were evaluated. After taking preoperative CT scans of the injured wrists, 3D images of the distal radius were created. Fracture reduction, implants choices, and placements simulation were performed based on the 3D images. One month after the surgery, postoperative CT images were taken. The reproducibility was evaluated with preoperative plan and postoperative 3D images. The images were compared with the three-dimensional coordinates of radial styloid process, volar and dorsal edges of sigmoid notch, and the barycentric coordinates of the three reference points. The reproducibility of the preoperative plan was evaluated by the distance of the coordinates between the plan and postoperative images for the reference points. The reproducibility of radial inclination and volar tilt on three-dimensional images were evaluated by intra-class correlation coefficient (ICC).

**Results:**

The distances between the preoperative plan and the postoperative reduction for each reference point were (1) 2.1±1.3 mm, (2) 1.9±1.2 mm, and (3) 1.9±1.2 mm, respectively. The distance between the preoperative plan and postoperative reduction for the barycentric coordinate was 1.3±0.8 mm. ICCs were 0.54 and 0.54 for the volar tilt and radial inclination, respectively (*P*<0.01).

**Conclusions:**

Three-dimensional preoperative planning for the osteosynthesis of distal radius fracture was reproducible with an error of about 2 mm for each reference point and the correlations of reduction shapes were moderate. The analysis method and reference points may be helpful to understand the accuracy of reductions for the three-dimensional preoperative planning in the osteosynthesis of distal radius fractures.

**Trial registration:**

Registered as NCT02909647 at ClinicalTrials.gov

## Background

Recently, the utility of computer-assisted orthopedic surgery (CAOS) has been widely reported [1, 2]. This technology refers to approaches that use computer-enabled simulations, tracking systems, or robotic devices to improve visibility of the surgical field and increase the surgical accuracy [3–5]. Evaluations of three-dimensional (3D) bone morphology, preoperative planning, and intraoperative navigation based on computer-aided technology are considered to be effective means to increase the accuracy of surgery and reduce complications. On the other hand, due to the complexity of preoperative planning and intraoperative registration procedures, and invasion of normal sites to obtain reference points, it is only applied by limited facilities and to certain surgeries [1].

Osteosynthesis for fractures is one of the most frequent surgical procedures in the orthopedic field [6–8]. In order to recover the lost motor function caused by fractures, it is important to achieve optimal reduction and appropriate internal fixation [9–12]. Suboptimal reduction/internal fixation causes complications such as delayed bone union, re-dislocation of fractures, and malunions. These complications prolong the patient’s functional recovery [9, 11, 13]. Prevention of these complications requires accurate reduction and repositioning according to individual fracture types and bone morphologies, and selection and placement of optimal implants. For this purpose, computer-assisted understanding of fracture patterns, reduction images, and prediction of internal fixation prior to surgery are helpful. However, due to the variety of fracture types and implant choices, the introduction of computer-aided technology for preoperative planning for fracture treatment has not yet been widely adopted.

In a previous study, a 3D preoperative planning system was developed to manage distal radius fractures [14]. This system allows visualization of the reduction process and implant placement/choices in a virtual space. 3D preoperative planning showed excellent reproducibility in terms of implant choices and placements in the osteosynthesis of distal radius fractures. However, there is no method to evaluate the reduction shape reproducibility of the preoperative plan three dimensionally. In this study, we developed a method to evaluate the reduction shape reproducibility based on three-dimensional coordinates of distal radius reference points. Using this method, the reproducibility of 3D preoperative planning with measurement indices based on the three-dimensional reference points was evaluated. We hypothesized 3D preoperative planning for the osteosynthesis of distal radius fracture would be reproducible with evaluations of three-dimensional reference points.

## Methods

This study protocol was approved by the Institutional Review Board. This was a case control study (level of evidence III). This study was registered as NCT02909647 at ClinicalTrials.gov. This study included the patients who underwent osteosynthesis with volar locking plate using 3D preoperative planning during the period from October 2015 to December 2019. The follow-up period was 6 months. Sixty-three wrists of 63 distal radius fracture patients (46 females, 17 males, mean age 65.1 years, age range 18-91) were evaluated. Written consent was obtained from all study participants. Patients under the age of 18, patients with bilateral distal radius fractures, and/or patients with a history of traumatic arm injury were excluded. We also excluded the patients who underwent osteosynthesis without using volar locking plate. All patients had CT images of the injured wrist taken before and 1 month after surgery. According to the preoperative X-ray (posterior-anterior and lateral view) and CT scans, fractures were classified using the AO classification system. CT images were taken with a tube setting of 120kV and 100mAS, a section thickness of 0.8 mm and a pixel size of 0.3×0.3 mm (Sensation Cardiac, Siemens). The CT images were taken from the metacarpal bone level to approximately 13 cm of the proximal forearm.

### 3D preoperative planning

**Fig. 1:**
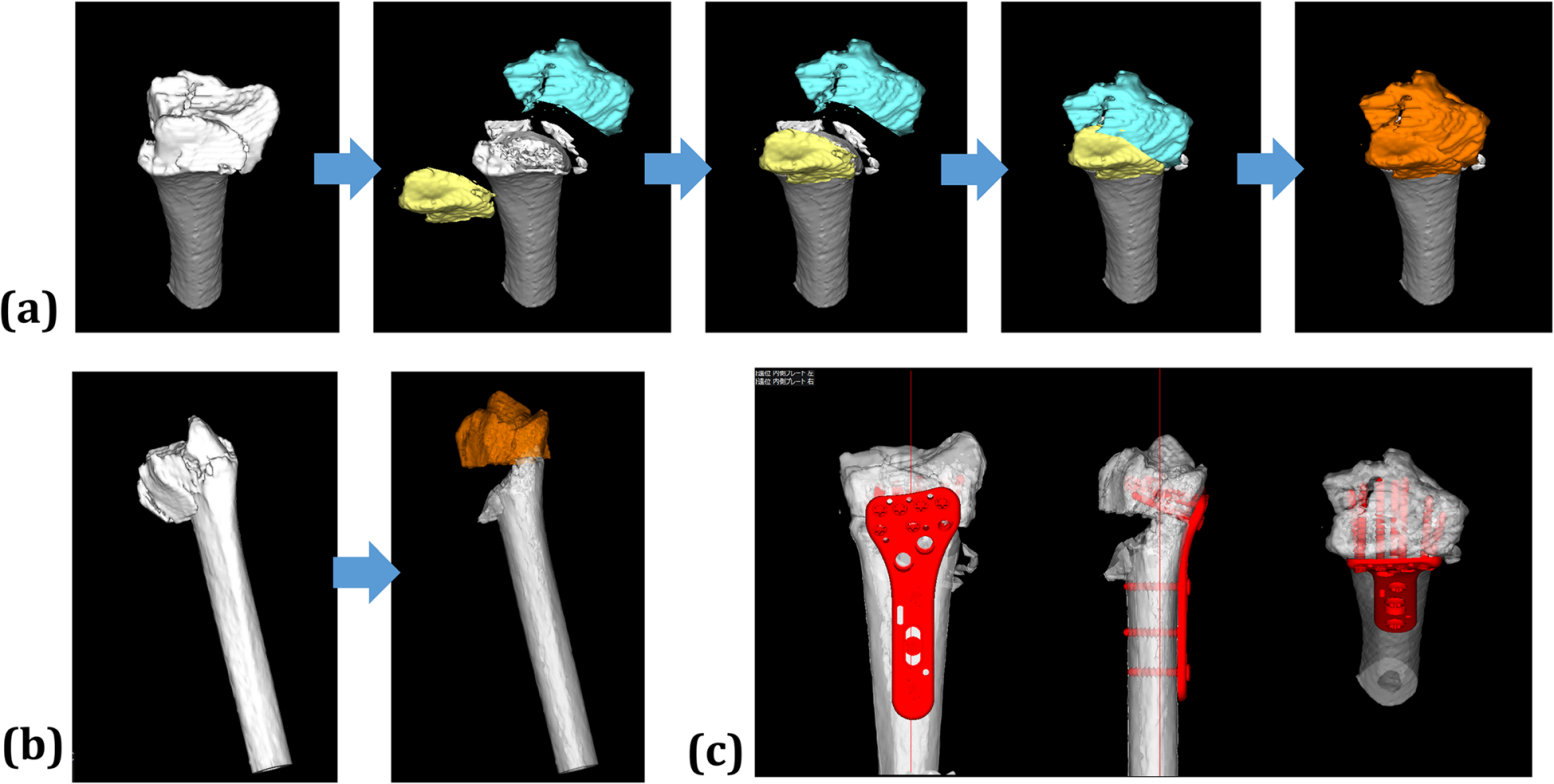
An example of the preoperative planning process. **a** Reduction simulation in the axial view, **b** reduction simulation in the sagittal view, **c** implant choices and placement

Before surgery, 3D preoperative planning and surgery simulation were performed (Fig. 1). Preoperative planning software (Zed-Trauma distal radius stage, LEXI Co., Ltd. Tokyo, Japan) was used for the reduction simulations and implant placements. After importing the DICOM images into the software, a 3D image of the distal radius was created. Each distal radius fracture was segmented according to the fracture fragments. Each fragment was repositioned in accordance with fracture lines. After repositioning the fragments, the 3D shape of the distal radius was checked. Fragment reductions were simulated to regain volar tilt, radial inclination, and radius shortening, with reductions in the gap/step-off for the articular surface. Bone fragments larger than 5 mm were taken into account for reduction, but those smaller than that were excluded from the reduction simulation. After the reduction, simulations of the volar locking plate implantation with various sizes of plates and screws were performed. Computer aided design models of different-sized implants were installed in the software. The plate size was chosen to cover the distal fragment maximally and not exceed the width of the distal radius. Screws with sufficient length for the anterior-posterior diameter of the distal radius were selected. The 75 to 100% of the radius anterior-posterior lengths along the screws were defined as the sufficient length. After the preoperative planning, osteosynthesis was performed under general anesthesia. The volar approach was applied in all cases. The reductions were performed according to the fracture lines visualized in the surgical site and with the assistance of fluoroscopic image. For the intra-articular fractures, the arthroscopic reduction of the articular surface or the removal of small fragments was performed. Bone fragments less than 5 mm in the joint were excised if they could not be fixed. If they could be fixed to fit on the joint surface, they were fixed with subchondral support. During the surgery, the surgeon performed the reduction and placement of the implants while comparing images between the preoperative plan and fluoroscopy during surgery. The distal screw holes were drilled with a drill guide until it reached the dorsal cortex. The screw lengths were measured and the same or 1-2 mm shorter screws as the measurement were selected with reference to the preoperative plan. If the dorsal cortex was comminuted and difficult to measure, the screws were selected according to the preoperative plan. The surgeries were performed by six trainees (residents and fellows) and one hand surgeon. The hand surgeon participated in all surgeries.

### 3D image analysis

**Fig. 2:**
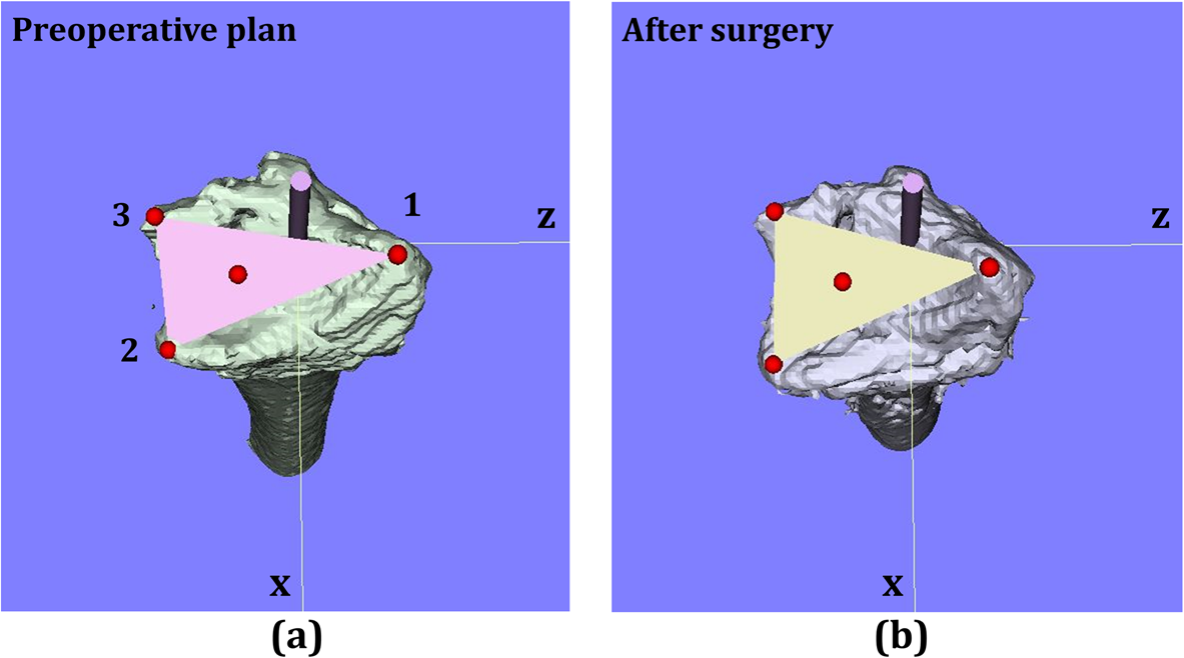
Axial view of 3D image. **a** Preoperative plan, **b** postoperative image. Three reference points, (1) radial styloid process, (2) sigmoid notch volar edge, and (3) sigmoid notch dorsal edge, were marked on the image. The barycentric coordinates of the plane connecting the three reference points were measured

**Fig. 3:**
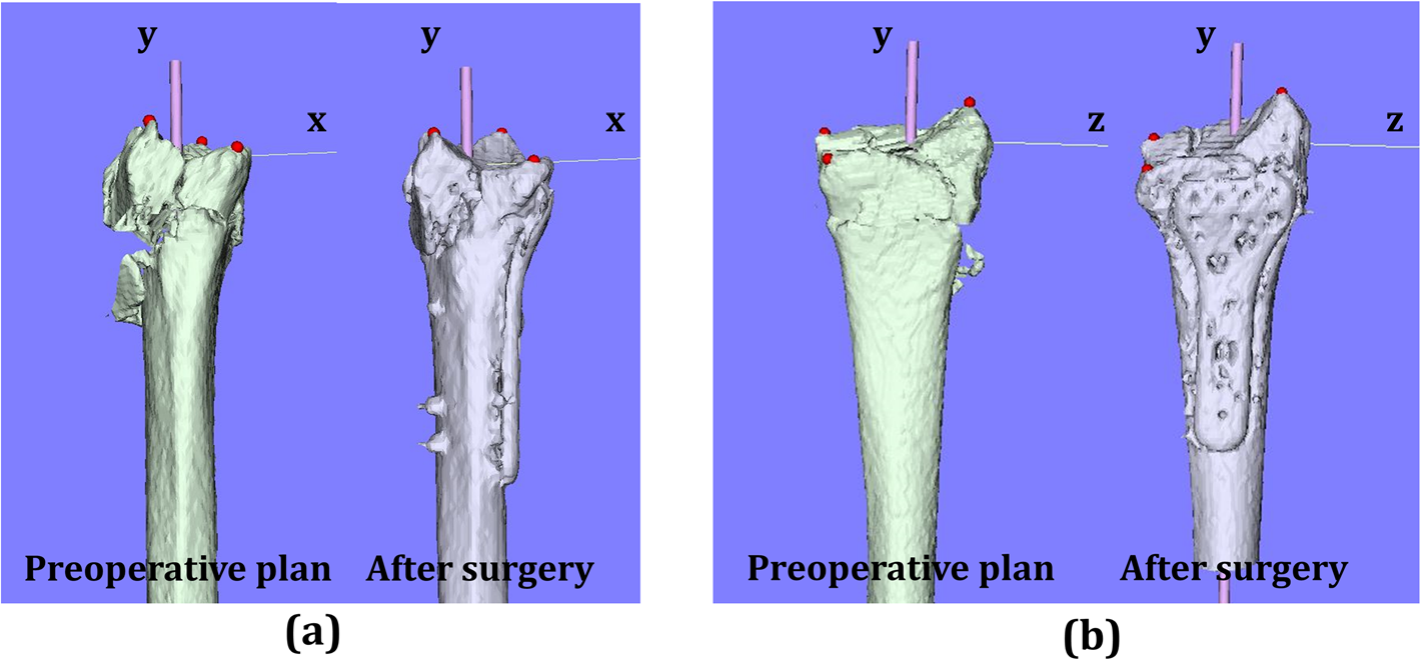
Sagittal and coronal views of a 3D image. **a** Sagittal view, **b** coronal view

Pre- and post-operative 3D images of the distal radius were analyzed with the image analysis software (BoneSimulater, Orthree, Osaka, Japan). After importing image data into the software, a three-dimensional surface model of the radius was constructed with a surface construction algorithm (Figs. 2 and 3). A coordinate system was constructed following the international society of biomechanics recommended protocols [15, 16]. The long axis of the radius was calculated from the three-dimensional surface model of the intact part of the preoperative distal radius image. Using the intact part of the distal radius image, image registration for the preoperative plan and postoperative reduction were performed. The y-axis was defined as the long axis of the radius, and the proximal direction was defined as positive. The *z* axis was parallel to the orthogonal projection of the line that initiated at the base of the distal ulnar sigmoid notch and continued to the radial styloid process on the plane perpendicular to the *y*-axis. The radial direction on the *z*-axis was defined as positive. The *x*-axis was normal to the yz plane and the palmar direction was defined as positive. The yz plane, xy plane, and xz plane were defined as the coronal plane, sagittal plane, and axial plane, respectively. The origin of coordinates was defined as the intersection of the joint surface and the radius long axis on the preoperative plan image. On the pre- and post-operative 3D images, three reference points, (1) radial styloid process, (2) sigmoid notch volar edge, and (3) sigmoid notch dorsal edge, were marked (Fig. 2a). The three-dimensional coordinates of each reference point and the barycentric coordinates of the plane connecting the three reference points were evaluated with the 3D images of the preoperative plan and postoperative reductions. In addition, the plane area connecting the three reference points was measured using the preoperative plan and postoperative reductions images.

The angle between a connecting line from reference point (2) to the reference point (3) and a line perpendicular to the longitudinal axis of the radius was measured as the volar tilt on a 3D image in the sagittal view. The angle between a line from reference point (1) to reference point (2) and a line perpendicular to the longitudinal axis of the radius was measured as the radial inclination on a 3D image in the coronal view.

For the evaluations of clinical outcomes, the Mayo wrist score was recorded at 3 and 6 months after the surgery. The Mayo wrist score evaluates the pain intensity, the active flexion/extension arc, grip strength, and the ability to return to regular employment or activities. Scores range from 0 to 100 with a lower score indicating a worse wrist condition and a higher score indicating a better wrist condition.

### Statistical analysis

The results are expressed as the mean (standard deviation). The average positions for the three reference points relative to the origin were analyzed for the preoperative plan and postoperative reduction. To test the normality of datasets, the Shapiro-Wilk test was used. The distances between the preoperative plan and postoperative reduction for each reference point were measured and compared among the reference points using one-way repeated measures of analysis of variance (ANOVA). The plane area connecting the three reference points and the Mayo wrist scores were compared between the preoperative plan and postoperative reduction using the paired *t* test. Correlations for radial inclination and volar tilt between the preoperative plan and postoperative reduction were analyzed with intra-class correlation coefficients (ICC). *P* values of less than 0.05 were considered significant. All analyses were performed using BellCurve for Excel version 2.12 (SSRI Co., Tokyo, Japan).

## Results

**Fig. 4:**
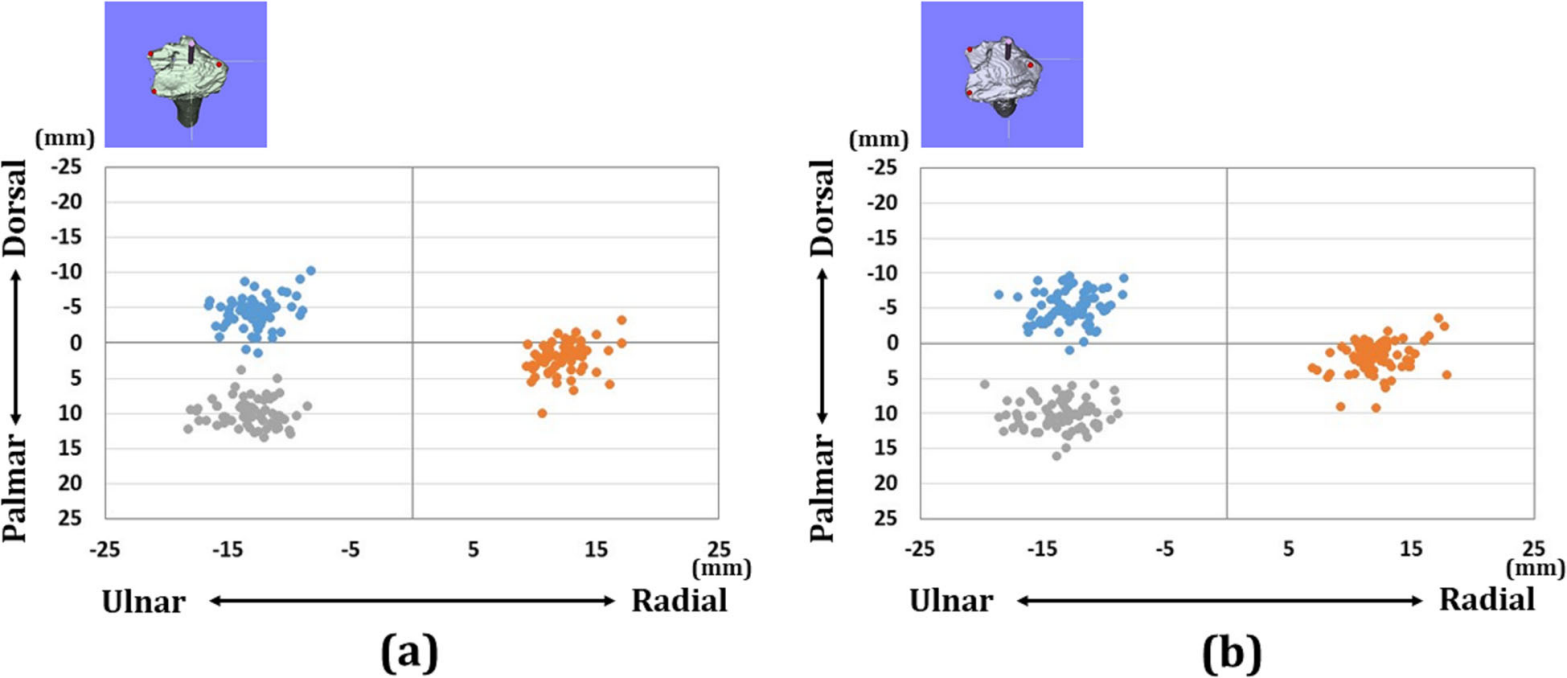
Results of coordinates for three reference points in the axial plane. **a** Results of coordinates for the preoperative plan image. **b** Results of coordinates for the postoperative reduction image. The orange dots indicate radial styloid process: reference point (1). The gray dots indicate sigmoid notch volar edge: reference point (2). The blue dots indicate sigmoid notch dorsal edge: reference point (3)

**Fig. 5:**
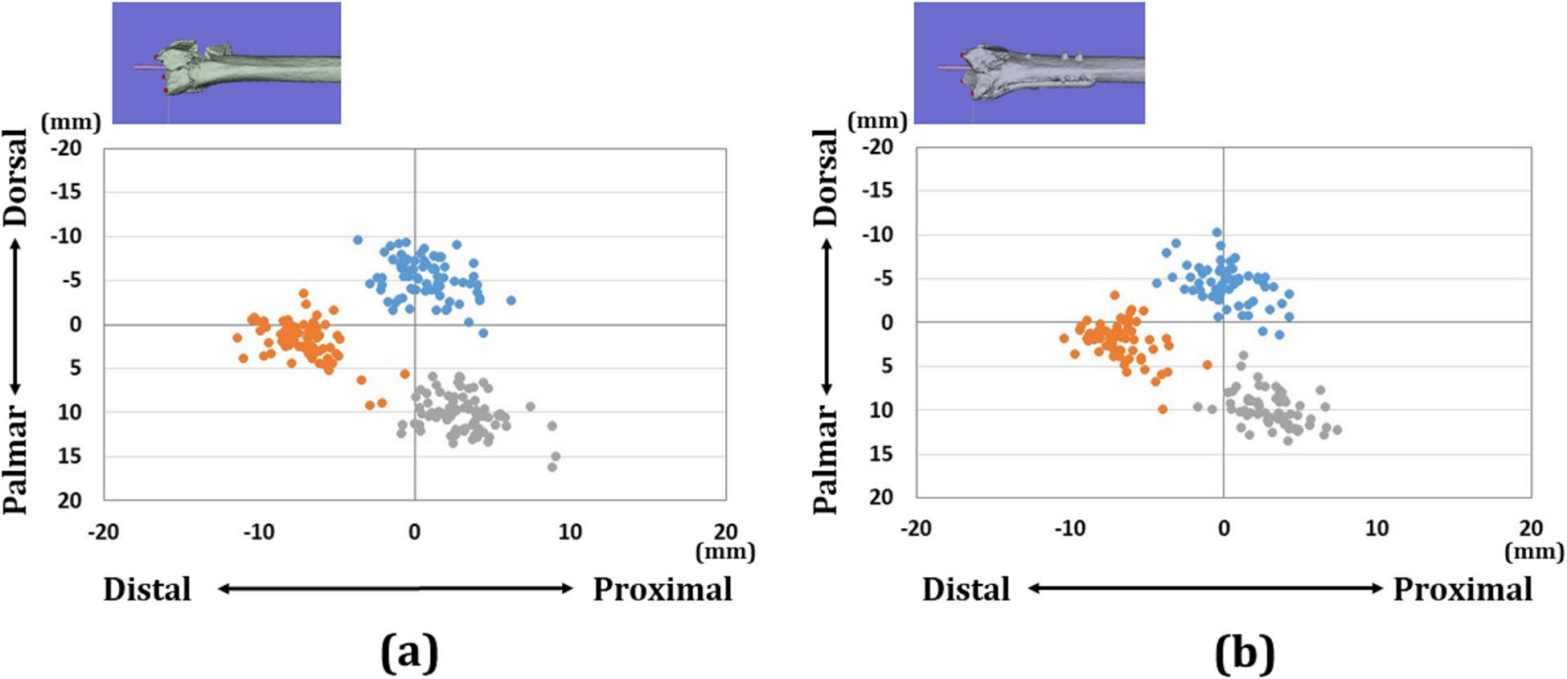
Results of coordinates for three reference points in the sagittal plane. **a** Results of coordinates for the preoperative plan image. **b** Results of coordinates for the postoperative reduction image. The orange dots indicate radial styloid process: reference point (1). The gray dots indicate sigmoid notch volar edge: reference point (2). The blue dots indicate sigmoid notch dorsal edge: reference point (3)

**Fig. 6:**
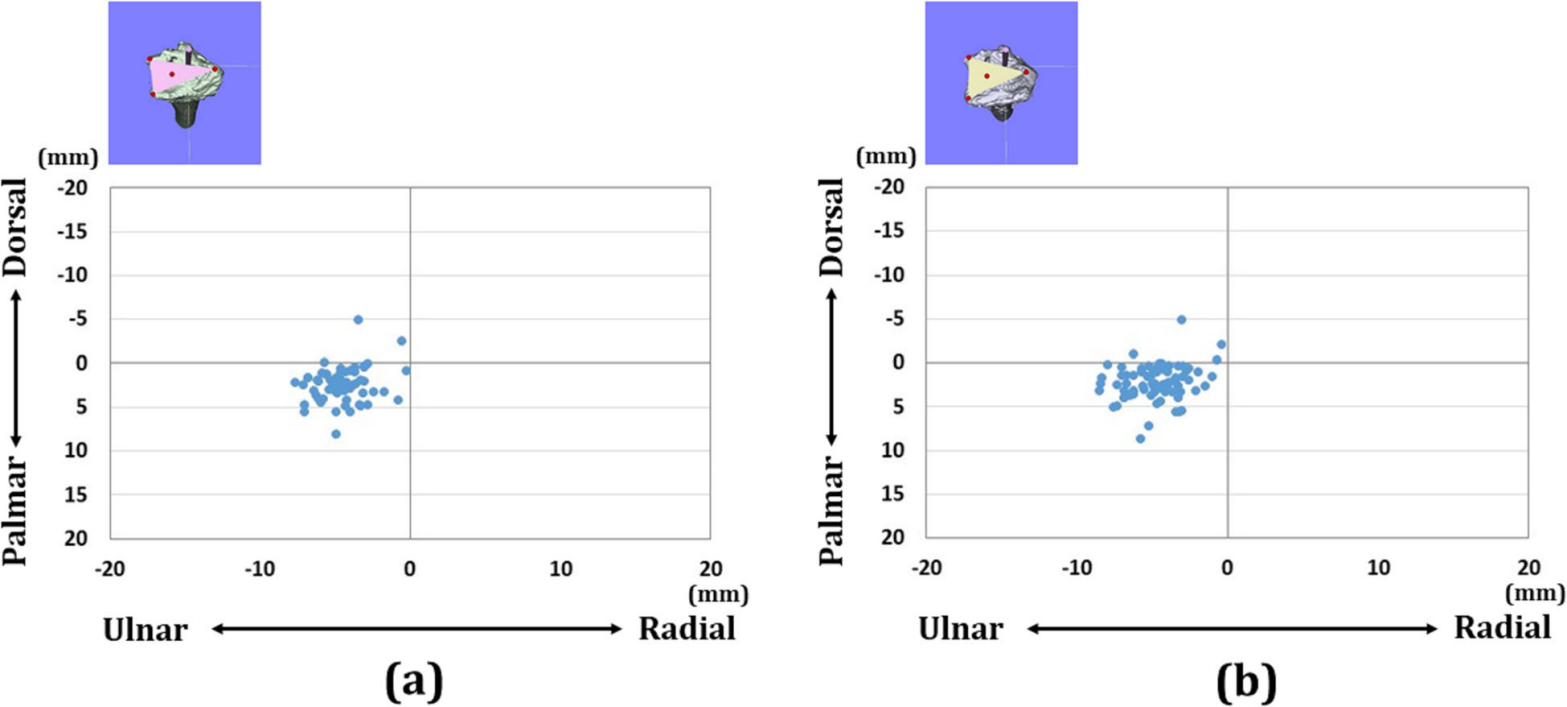
Results for the barycentric coordinates in the axial plane. **a** Results of coordinates for the preoperative plan image. **b** Results of coordinates for the postoperative reduction image

There were 18 wrists with A3 fractures, 25 wrists with C2 fractures, and 20 wrists with C3 fractures in each group. Forty-four wrists were displaced in the dorsal direction and 19 wrists were displaced in the palmar direction. The distribution of each reference point in the axial and sagittal planes is shown in Figs. 4, 5, and 6. In the preoperative plan, each reference point was located (1) 14.4 (SD 1.6) mm radial-dorsal-distal position, (2) 16.9 (SD 2.1) mm ulnar-palmar-proximal position, and (3) 13.6 (SD 1.6) mm ulnar-dorsal-proximal position to the origin. The barycentric coordinate was located at a 5.8 (SD 1.5) mm ulnar-palmar-distal position to the origin. After surgery, each reference point was located (1) 14.4 (SD 1.9) mm radial-palmar-distal position, (2) 17.3 (SD 2.5) mm ulnar-palmar-proximal position, and (3) 14.1 (SD 2.0) mm ulnar-dorsal-proximal position to the origin. The barycentric coordinate was located at a 5.9 (SD 1.8) mm ulnar-palmar-distal position to the origin. The distances between the preoperative plan and postoperative reduction for each reference point were (1) 2.1 (SD 1.3) mm, (2) 1.9 (SD 1.2) mm, and (3) 1.9 (SD 1.2) mm, respectively. The distance between the preoperative plan and postoperative reduction for the barycentric coordinate was 1.3 (SD 0.8) mm. The distance was significantly smaller in the barycentric coordinates compared to the other reference points (*P*<0.05).

The plane areas connecting the three reference points were 191.6 (SD 28.3) mm^2^ and 203.8 (SD 37.3) mm^2^ for the preoperative plan and postoperative reduction, respectively. There was a significantly larger plane area in the postoperative reduction image compared to the pre-operative plan image (*P*<0.01).

**Fig. 7:**
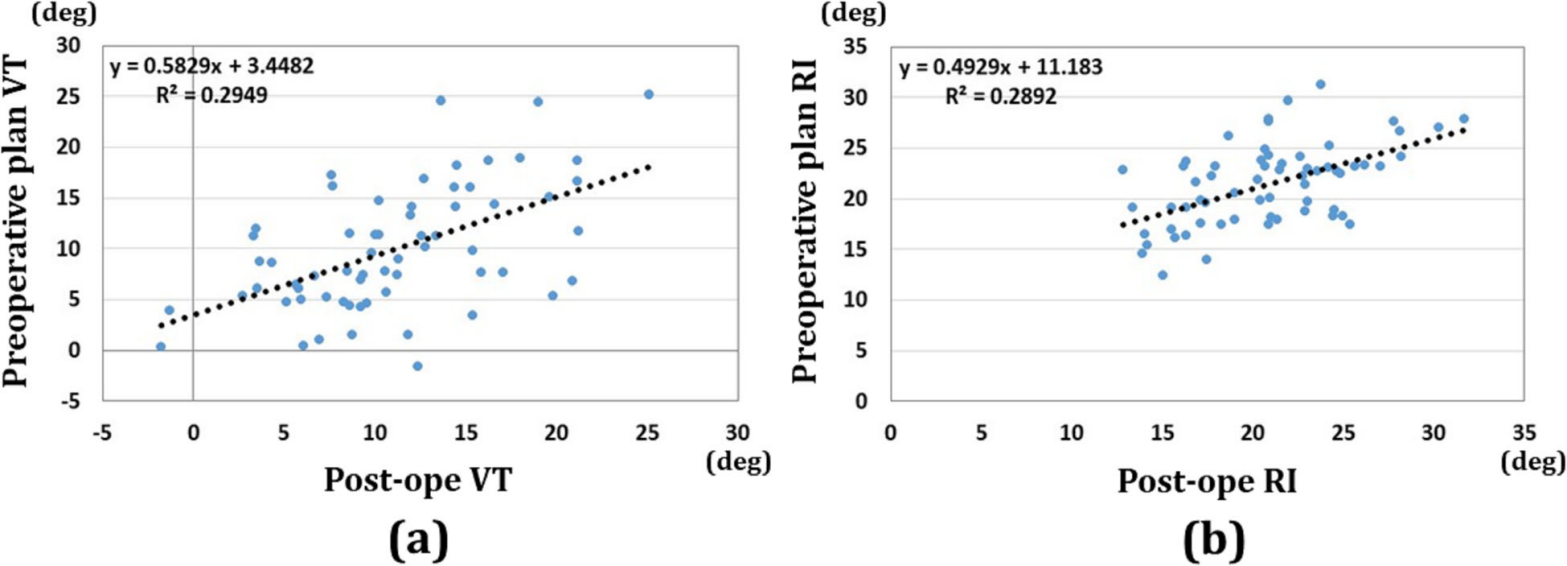
Results of correlations for the volar tilt and radial inclination. **a** Results of volar tilt correlations between the preoperative plan and postoperative reduction. **b** Results of radial inclination correlations between the preoperative plan and postoperative reduction

The results of correlations for volar tilt and radial inclination are shown in Fig. 7. The volar tilts were 10.9 (SD 5.5) degrees and 9.3 (SD 6.0) degrees for the preoperative plan and postoperative reduction, respectively. The radial inclinations were 20.8 (SD 4.4) degrees and 21.5 (SD 4.0) degrees for the preoperative plan and postoperative reduction, respectively. The correlation between the preoperative plan and postoperative reduction for the volar tilt was 0.54 (*P*<0.01), while the correlation between the preoperative plan and postoperative reduction for the radial inclination was 0.54 (*P*<0.01).

The Mayo wrist scores were 65.3 (SD 12.8) points and 77.2 (SD 8.9) points at 3 and 6 months after the surgery. In 3 months after the surgery, the average grip strengths were 15.8 (SD 7.3) kgw and 24.9 (SD 9.0) kgw for the affected side and unaffected side, respectively. The averages of wrist flexion/extension arc were 98.6 (SD 24.7) degrees and 122.3 (SD 26.7) degrees for the affected side and unaffected side, respectively. In 6 months after surgery, the average grip strengths were 19.3 (SD 7.8) kgw and 24.9 (SD 9.4) kgw for the affected side and unaffected side, respectively. The averages of wrist flexion/extension arc were 108.8 (SD 14.7) degrees and 119.2 (SD 14.9) degrees for the affected side and unaffected side, respectively. There were significant differences in the grip strength and wrist flexion/extension arc between affected side and unaffected side at both 3 and 6 months (*P*<0.01).

## Discussion

The technology for CAOS mainly helps clinicians to improve and manage complete surgical procedures. These surgical procedures include preoperative analysis and decisions, intraoperative procedures, and postoperative management. Individual technologies include virtual surgical planning, computer-assisted navigation within an operation, virtual operation training, etc. [2]. Recently, there has been increased interest in using computer-assisted technologies in orthopedic trauma cases. Preoperative surgery using 3D printing models or surgical simulation may be beneficial to confirm the reduction and fixation procedures [5, 17, 18]. It has been reported that 3D printing models effectively reduced the surgical time, and provided more effective communication between doctors and patients [18]. Although visibility of fractures and surgical plans were improved with computer-assisted technologies, their clinical significance and reproducibility have not yet been fully confirmed.

This study sought to determine the accuracy of 3D digital preoperative planning for the osteosynthesis of distal radius fractures. Visualization of morphology for the 3D reduction shape is one of the benefits of the virtual simulation of osteosynthesis. After the introduction of CT scans, distal radius fracture patterns have become well recognized in terms of their 3D shapes [19, 20]. In most cases, conventional preoperative planning by transferring X-ray images with tracing paper is still being used. 3D preoperative planning for osteosynthesis has advantages in terms of visualizing the reduction shape and implants choices, as well as sharing this information with assistants and other medical staff. The method described in this study can be performed using software on a computer. The user can learn how to operate the software by performing several trials. After learning the simulation method, normally the simulation can be done in 15 to 30 min. If there is a computer which installed these programs, it will be usable in any place. In our previous study, it was shown that there was excellent reproducibility for implant choices [14]. However, the reproducibility of the 3D shape of reductions for preoperative planning was unclear. This was because there was no method to evaluate the reproducibility of the 3D shape. In this study, we developed a method to analyze the 3D reduction shape by registering 3D images in the preoperative plan and postoperative reduction. As a result, it was found that 3D preoperative planning was reproducible with an error of about 2 mm for each reference point. The barycentric coordinates showed better reproducibility compared to the other reference points. The correlations of volar tilt reduction parameters and radial inclination were moderate. Although several reports evaluated the reduction accuracy of a 3D plan and guided osteotomy for distal radius malunions [21–23], there are few reports that have evaluated the three-dimensional reproducibility of preoperative planning in the osteosynthesis of acute distal radius fractures. According to reports on malunions, the difference between the preoperative plan and postoperative reduction was about 1-2 mm. The average error for the reference points in this study was slightly larger than in these corrective osteotomy studies. Although the reduction parameters were within the range of the general criteria for distal radius fracture reduction [24], there may be room to improve the accuracy of reductions for reference points. This method and the reference points may be useful in understanding the three-dimensional reproducibility of preoperative planning in the osteosynthesis of distal radius fractures.

There are several limitations to this study. 3D preoperative planning requires a CT scan. CT has clear advantages in terms of excellent bone–soft tissue contrast and no geometrical distortion, although its acquisition exposes the patient to radiation; care is needed to reduce radiation exposure. According to one previous study [25], it was possible to evaluate bone morphology with high accuracy even if the radiation exposure dose was 1/30 the level of a standard CT scan. This may be one solution to the radiation exposure problem. Second, we did not compare the reduction shape with the unaffected side of the patient’s wrist. Comparing the reduction position with the unaffected side of the wrist may be better to evaluate the reduction accuracy. Now, we are adjusting the protocol to take the bilateral wrist CT scans with low dose radiation. Third, we did not compare the results with the reproducibility of reduction in cases without 3D preoperative planning. This is because evaluations of reduction based on 3D reference points were possible only when performing 3D preoperative planning. Another limitation is the moderate reproducibility for the reduction shape. There were also significant differences in the plane area connecting the three reference points between the preoperative plan and postoperative reduction. This was because the articular surface gap may not have been reduced sufficiently as estimated in the preoperative planning. The reconstruction of the joint surface shape requires more precise reduction during surgery.

In conclusion, three-dimensional preoperative planning for the osteosynthesis of distal radius fractures was reproducible, with an error of about 2 mm for 3D reference points, and the reduction shape correlations were moderate. The analysis method and reference points described in this study may be helpful to understand the accuracy of reductions during the preoperative three dimensional planning of osteosynthesis in distal radius fractures. It may be necessary to improve the accuracy of the reduction by developing a method to compare the preoperative image with the intraoperative fluoroscopic image.

## Data Availability

The datasets used and/or analyzed during the current study are available from the corresponding author on reasonable request.

## Abbreviations

CAOS: Computer-assisted orthopedic surgery
3D: Three dimensional
CT: Computed tomography
ANOVA: Analysis of variance
ICC: Intra-class correlation coefficients
